# The effect of higher or lower mean arterial pressure on kidney function after cardiac arrest: a post hoc analysis of the COMACARE and NEUROPROTECT trials

**DOI:** 10.1186/s13613-023-01210-0

**Published:** 2023-11-21

**Authors:** Johanna Laurikkala, Koen Ameloot, Matti Reinikainen, Pieter-Jan Palmers, Cathy De Deyne, Ferdinande Bert, Matthias Dupont, Stefan Janssens, Joseph Dens, Johanna Hästbacka, Pekka Jakkula, Pekka Loisa, Thomas Birkelund, Erika Wilkman, Suvi T. Vaara, Markus B. Skrifvars

**Affiliations:** 1 Department of Anaesthesiology, Intensive Care and Pain Medicine, Helsinki University Hospital and University of Helsinki, Haartmaninkatu 9, 00290 HUS Helsinki, Finland; 2 Department of Cardiology, Ziekenhuis Oost-Limburg, Genk, Belgium; 3 Departement de Cardiologie/Soins Intensifs Adultes, CHC–Montlégia, Liège, Belgique; 4 Department of Cardiology, University Hospitals Leuven, Leuven, Belgium; 5 Faculty of Medicine and Life Sciences, University Hasselt, Diepenbeek, Belgium; 6 Department of Anaesthesiology and Intensive Care, University of Eastern Finland and Kuopio University Hospital, Kuopio, Finland; 7 Department of Anesthesiology and Critical Care Medicine, Ziekenhuis Oost-Limburg, Genk, Belgium; 8 Department of Anesthesia and Intensive Care, Tampere University Hospital and Tampere University, Tampere, Finland; 9 Department of Intensive Care, Päijät-Häme Central Hospital, Lahti, Finland; 10 Aarhus University Hospital, Aarhus, Denmark; 11 Department of Emergency Care and Services, Helsinki University Hospital and University of Helsinki, Helsinki, Finland

**Keywords:** Acute kidney injury, Kidney Disease Improving Global Outcomes, Out-of-hospital cardiac arrest

## Abstract

**Background:**

We aimed to study the incidence of acute kidney injury (AKI) in out-of-hospital cardiac arrest (OHCA) patients treated according to low-normal or high-normal mean arterial pressure (MAP) targets.

**Methods:**

A post hoc analysis of the COMACARE (NCT02698917) and Neuroprotect (NCT02541591) trials that randomized patients to lower or higher targets for the first 36 h of intensive care. Kidney function was defined using the Kidney Disease Improving Global Outcome (KDIGO) classification. We used Cox regression analysis to identify factors associated with AKI after OHCA.

**Results:**

A total of 227 patients were included: 115 in the high-normal MAP group and 112 in the low-normal MAP group. Eighty-six (38%) patients developed AKI during the first five days; 40 in the high-normal MAP group and 46 in the low-normal MAP group (*p* = 0.51). The median creatinine and daily urine output were 85 μmol/l and 1730 mL/day in the high-normal MAP group and 87 μmol/l and 1560 mL/day in the low-normal MAP group. In a Cox regression model, independent AKI predictors were no bystander cardiopulmonary resuscitation (*p* < 0.01), non-shockable rhythm (*p* < 0.01), chronic hypertension (*p = *0.03), and time to the return of spontaneous circulation (*p* < 0.01), whereas MAP target was not an independent predictor (*p* = 0.29).

**Conclusion:**

Any AKI occurred in four out of ten OHCA patients. We found no difference in the incidence of AKI between the patients treated with lower and those treated with higher MAP after CA. Higher age, non-shockable initial rhythm, and longer time to ROSC were associated with shorter time to AKI.

*Clinical trial registration*: COMACARE (NCT02698917), NEUROPROTECT (NCT02541591).

**Supplementary Information:**

The online version contains supplementary material available at 10.1186/s13613-023-01210-0.

## Introduction

After successful resuscitation from cardiac arrest (CA), the overall mortality rate relates mainly to hypoxic–ischaemic brain injury. However, damage to other organs also has prognostic implications [1, 2]. Acute kidney injury (AKI) is one feature of post-cardiac arrest syndrome [1, 3–7]. The incidence of AKI after CA varies from 37 to 81% [3]. Circulatory arrest and subsequent return of spontaneous circulation (ROSC) result in ischaemia–reperfusion injury. During post-CA syndrome, possible causes of renal damage are direct renal ischaemic–reperfusion injury and indirect renal damage characterized by the activation of inflammatory and coagulation pathways leading to renal dysfunction [3, 6]. Another important cause for AKI after CA is post-resuscitation myocardial dysfunction leading to hemodynamic failure, hypoperfusion, and even post-resuscitation cardiogenic shock and worsening renal function [6–10].

AKI denotes a sudden reduction in kidney function manifested by a rise in serum creatinine levels and reduced urine output (UO). Studies have suggested an association between AKI severity and outcomes after OHCA [6]. Various criteria have been used to define AKI, the Kidney Disease Improving Global Outcomes (KDIGO) published in 2012 being the currently used tool [11–14].

In the multicentre randomized COMACARE and Neuroprotect pilot trials, adult comatose survivors of OHCA were treated with high-normal or low-normal MAP targets.[15–18] The aim of the current analysis was to examine the effect of different MAP levels on the incidence of AKI after OHCA. We calculated the AKI KDIGO stages using both creatinine and UO data for the patients over 5 days and analysed the incidence of AKI in the high-normal and the low-normal MAP groups.

## Methods

### Setting and participants

This is a post hoc analysis of two randomized clinical trials: the COMACARE (Carbon dioxide, Oxygen and Mean arterial pressure After Cardiac Arrest and Resuscitation) and the Neuroprotect (Neuroprotective Goal Directed Hemodynamic Optimization in Post-Cardiac Arrest Patients) trials [15, 17] The details of these studies are published earlier [15, 17]. Briefly, six intensive care units (ICU) in Finland and one in Denmark participated in the COMACARE trial conducted from March 2016 to March 2017, and two tertiary care hospitals in Belgium participated in the Neuroprotect trial conducted from July 2015 to May 2018. Both trials were approved by the local ethics committees. The participants were adult patients resuscitated from witnessed OHCA, with slight differences in the inclusion and exclusion criteria between the two trials (Additional file 7: Table S1). In the COMACARE trial, OHCA patients with shockable rhythms were randomized to two groups targeting either an MAP of 65–75 mmHg or 80–100 mmHg. In the Neuroprotect trial, OHCA patients with shockable or non-shockable rhythms were randomized into MAP targets of 65 mmHg or 85–100 mmHg. In the COMACARE trial, fluid boluses and norepinephrine infusions were used, when needed, to reach the MAP target, and if cardiac output was low, the use of inotropes was also allowed. Any treatment of severe hypertension was up to the treating clinician. In the Neuroprotect trial, cardiac output was monitored and mixed venous oxygen saturation was targeted to 65–75% with norepinephrine, dobutamine, and fluids. All COMACARE participants received targeted temperature management, targeting 33 ℃ or 36 ℃ for 24 h. In the Neuroprotect trial, all patients were treated with a temperature target of 33 ℃ for 24 h. All patients in both trials received standard care, monitoring, and assessment, including invasive blood pressure monitoring and daily UO measurement.

### Data collection

We collected data on patient demographics, pre-existing chronic diseases, CA, and resuscitation factors and immediate hospital management. The plasma creatinine values were collected on ICU admission and daily thereafter during the entire ICU stay. The patients had urinary catheters for the measurement of UO.

### Acute kidney injury by AKI KDIGO classification and study outcomes

We studied the incidence of AKI, which was defined as the occurrence of any AKI KDIGO stage (1–3) during the first five days after the OHCA. We analysed the cumulative development of AKI KDIGO stages 1–3 over time during the first five days and 48 h in the ICU and compared the severity of AKI by analysing creatinine and UO between the low-normal and high-normal MAP groups. We estimated baseline renal function using the Modification of Diet in Renal Disease (MDRD) for creatinine, assuming a glomerular filtration rate of 75 mL/minute/1.73 m^2^ [11]. In addition, we also estimated AKI with KDIGO but with the measured admission creatinine level as the baseline. Finally, due to the lack of hourly urine output, we used a modified definition of AKI KDIGO classification using daily UO averaged over 24 h for classes 2 and 3. The AKI KDIGO classes were defined as follows:

- Stage 1: a 1.5- to 1.9-fold increase in creatinine compared to the baseline creatinine or an absolute increase of more than 26.5 µmol/l within 48 h.
- Stage 2: a 2.0- to 2.9-fold increase in creatinine compared to baseline or in the definition including UO an average UO less than 0.5 ml/kg/hour per day.
- Stage 3: a threefold increase in creatinine compared to the baseline or an increase in to more than 353.6 µmol/l or in the definition including UO an average UO less than 0.3 ml/kg/hour per day.

### Assessment of acute kidney injury by creatinine clearance

We studied the cumulative development of AKI by analysing creatinine clearance values estimated using the 2021 Chronic kidney disease- Epidemiology Collaboration (CKD- EPI) equation [19–21]. We estimated daily glomerular filtration rate with CKD-EPI equation, taking into account serum creatinine concentration (μmol/ml), age and sex [19]. CKD is generally defined as GFR less than 60 ml/minute/1.73 m^2^ and we used the same definition for AKI [19].

### Statistical analysis

The categorical data are presented as counts (percentages), and the continuous data are shown as medians with interquartile ranges (IQR) or means with 95% confidence intervals. Comparisons were made using the Chi-square test or Mann–Whitney test when appropriate. Time to AKI was analysed using Cox proportional hazards regression, with the results reported as hazard ratios (HR) (95% confidence interval [22]). First, we entered age, no bystander CPR, non-shockable initial rhythm, hypertension, time to ROSC and the high-MAP target into five separate unadjusted models: (1) time to AKI of any severity (AKI KDIGO 1–3), (2) time to severe AKI (AKI KDIGO 2–3), (3) time to AKI of any severity (AKI KDIGO 1–3) when admission creatinine was used as baseline, (4) time to AKI of any severity (AKI KDIGO 1–3) when modified AKI KDIGO classification was based on creatinine and the average daily UO, and (5) time to AKI of any severity (AKI KDIGO 1–3) during the first 48 h after OHCA. To predict AKI, we constructed multivariate Cox models adjusted for age, no bystander CPR, non-shockable initial rhythm, hypertension, time to ROSC, and the MAP target. We used the Kruskal–Wallis test to compare distributions between more than two groups. In addition, we calculated days alive without AKI during the first five days in the ICU and compared these between the intervention groups. Furthermore, we compared the results obtained in the multivariable Cox models with cause-specific competing risk models, since traditional Cox regression might not accurately estimate the role of a particular risk when competing events, such as mortality exist [23, 24]. Finally, we divided the median noradrenalin dose over the first 36 h into two groups (higher or equal to the median dose or lower than the median dose) separately in the low- and high-MAP intervention groups. We compared to the prevalence of AKI based on the median noradrenaline dose in the low- and high-MAP groups and compared these with the Chi-square test. Competing risk analysis were performed with R software version 4.3.1 using survival package [25, 26]. The other statistical analyses were performed using SPSS 27 (IBM, Armonk, NY, USA), or GraphPad PRISM (version 7.0d for Mac OSX, GraphPad Software, La Jolla, CA, USA). A two-sided p value < 0.05 was considered statistically significant.

## Results

**Fig. 1:**
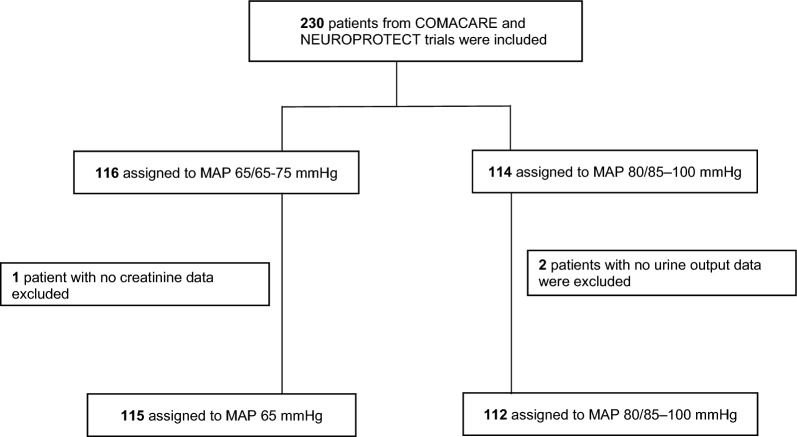
Flowchart of study patients

**Fig. 2:**
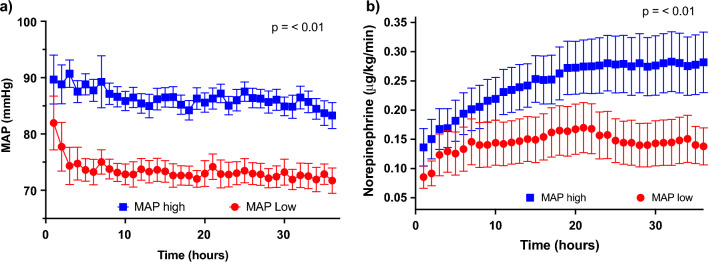
Comparison of MAP and norepinephrine use in patients randomized to either a high (80–100 mmHg) or low (> 65 mmHg) MAP target

A total of 230 patients from COMACARE and Neuroprotect trials were included. After excluding the patients without creatinine or UO data (*n* = 3), we included 227 patients in this study, 115 in the low-normal MAP group and 112 in the high-normal MAP group (Fig. 1). Patients randomized to the high-MAP treatment groups received significantly higher doses of norepinephrine (*p* < 0.01) and the MAP levels over time were significantly higher (Fig. 2).

### Incidence and risk factor for AKI

Using MDRD creatinine as the baseline, the incidence of AKI was 58% (*n* = 62) in the Neuroprotect trial and 20% (*n* = 24) in the COMACARE trial. In the total sample of 86 patients (38%) with AKI, AKI KDIGO 1 occurred in 46 (20%), AKI KDIGO 2 in 21 (9%), and AKI KDIGO 3 in 19 (8%) patients. When admission creatinine was used as baseline, 71 patients developed AKI with AKI KDIGO 1 in 55 (25%) patients, AKI KDIGO 2 in 7 (3%) patients, and AKI KDIGO 3 in 9 (4%) patients. Differences in patient characteristics, CA, and resuscitation factors and ICU care between the patients with AKI and those without are presented in Table 1. We also compared the patient characteristics between patients with three AKI KDIGO stages, 0, 1 and 2–3 (Additional file 8: Table S2). Sex (*p* = 0.03), age (*p* < 0.01), bystander CPR (*p* < 0.01), initial rhythm (*p* < 0.01), and hypertension (*p* < 0.01) were significantly different between the patients in the three AKI KDIGO groups.

**Table 1:** Patient characteristics of patients who developed acute kidney injury (KDIGO classes 1–3 defined by changes in creatinine) during the first five days after cardiac arrest

	Data available	All patients	Any AKI *n* = 86	No AKI *n* = 141	*p* value
Sex, n (%) (male)	227	176 (78)	64 (74)	112 (79)	0.38
Age, years (IQR)	227	63 (54–72)	67 (58–77)	61 (52–68)	** < 0.01**
BMI (IQR)	209	23 (21–25)	23 (22–26)	23 (21–25)	0.10
Cardiac arrest characteristics and resuscitation factors					
Bystander CPR or compressions only, n (%)	221	159 (72)	46 (55)	113 (83)	** < 0.01**
Public place, n (%)	223	111 (50)	38 (44)	73 (53)	0.19
Initial rhythm	226				
VF, n (%)		183 (81)	58 (67)	125 (89)	** < 0.01**
VT, n (%)		7 (3)	3 (4)	4 (3)	
PEA, n (%)		7 (3)	5 (6)	2 (1)	
ASY, n (%)		29 (13)	20 (23)	9 (6)	
Time to ROSC, minutes (IQR)	222	20 (14–25)	20 (15–30)	18 (14–25)	0.10
Medical history					
HTA, n (%)	221	106 (48)	51 (61)	55 (40)	** < 0.01**
Diabetes, n (%)*	104	10 (10)	9 (15)	1 (2)	**0.03**
COPD/asthma, n (%)	226	20 (9)	12 (14)	8 (6)	0.48
Norepinephrine at admission, mean (IQR)	209	0.13 (0.10–0.24)	0.15 (0.10–0.25)	0.13 (0.09–0.23)	0.86
Treatment					
CAG, n (%)	223	187 (84)	69 (81)	118 (86)	0.40
PCI, n (%)	224	112 (50)	42 (49)	70 (50)	0.89
MAP treatment group	227				0.51
High-normal, n (%)		112 (49)	40 (36)	72 (64)	
Low-normal, n (%)		115 (51)	46 (40)	69 (60)	
Duration of mechanical ventilation, days (IQR)	195	4 (2–7)	5 (3–8)	4 (2–6)	**0.01**
RRT, n (%)	226	3 (1.7)	3 (3.5)	0 (0)	**0.03**
Length of stay in ICU, days (IQR)	221	6 (4–9)	6 (4–12)	5 (3–8)	0.10
Death in ICU, n (%)	227	91 (40%)	52 (61%)	39 (28%)	** < 0.01**
Death in hospital, n (%)*					
(Neuroprotect trial)	106	106 (47%)	42 (68%)	20 (32%)	** < 0.01**
Mortality 30d, n (%)	227	93 (41%)	53 (62%)	33 (23%)	** < 0.01**
CPC 6 months, poor, n (%)	227	106 (47)	59 (69)	47 (33)	** < 0.01**

A two-sided *p* value < 0.05 was considered statistically significant (in bold)

The numbers are median (interquartile range), mean (95% CI), or n (%). BMI body mass index (kg/m^2^), CPR cardiopulmonary resuscitation, VF ventricular fibrillation, VT ventricular tachycardia, PEA pulseless electrical activity, ASY asystole, ROSC return of spontaneous circulation, HTA arterial hypertension, COPD chronic obstructive pulmonary disease, ACE angiotensin-converting enzyme, CAG coronary angiography, PCI percutaneous coronary intervention, SOFA sequential organ failure assessment, MAP mean arterial pressure, ICU intensive care unit, CPC cerebral performance category, RRT Renal replacement therapy

^*^”Diabetes mellitus and Death in hospital” data were available only in Neuroprotect trial

### AKI and mean arterial pressure treatment group

**Fig. 3:**
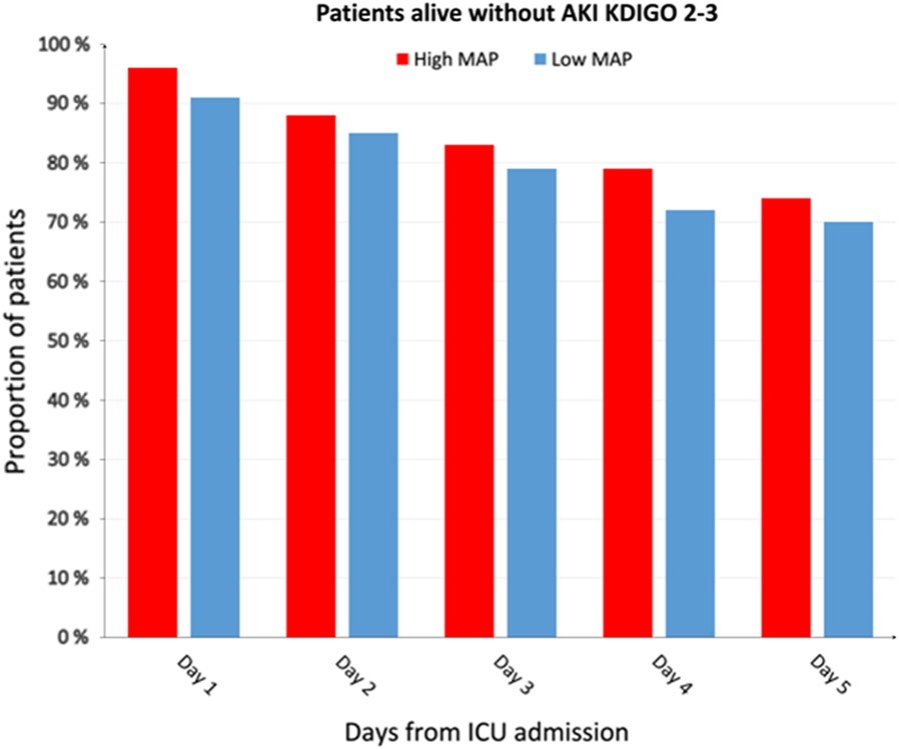
Cumulative percentage of alive patients without acute kidney injury defined as AKI KDIGO 2–3 during the first 5 days after out-of-hospital cardiac arrest. MAP mean arterial pressure

**Fig. 4:**
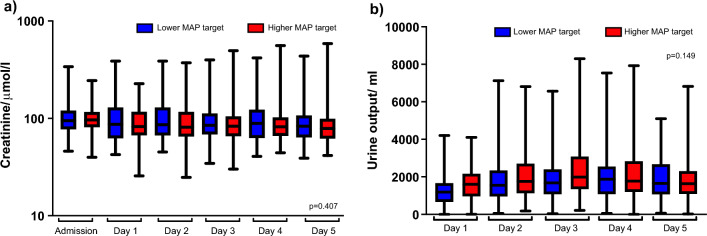
Kidney function over time in the high-normal and low-normal MAP groups by creatinine level (**a**) and the average daily urine output (**b**)

Eighty-six (38%) patients developed AKI during the first five days; 40 (36%) in the high-MAP group and 46 (40%) in the low-normal MAP group (p = 0.51) (Table 1). The cumulative number of patients without AKI KDIGO 2–3 and who are alive during the first five days after OHCA in the high-normal and low-normal MAP groups is shown in Fig. 3. The median creatinine and median daily UO were 85 (IQR 69–108) μmol/l and 1730 (IQR 1310–2350) mL/day in the high-normal MAP group and 87 (IQR 68–131) μmol/l and 1560 (IQR 1060–2030) mL/day in the low-normal MAP group. When comparing the creatinine level or UO between the high-normal and low-normal MAP groups, we found no statistically significant differences (Fig. 4). We repeated this analysis separately for the two included trials and found comparable results (Additional file 1: Figure S1).

Forty-one (38%) patients developed AKI during the first five days when CKD-EPI was used: 41 (38%) in the high-MAP group and 44 (38%) in the low-MAP group (*p* = 0.72). When comparing creatinine clearance between the high-normal and low-normal MAP groups, we found no statistically significant differences (Additional file 5: Figure S5). The median dose of noradrenalin over 36 h was 0.08 µg/kg/min in the low-MAP group and 0.2 µg/kg/min in the high-MAP group. The percentage and count of patients with AKI KDIGO 0, 1, and 2–3 treated with low or high doses of norepinephrine in the low-normal and high-normal MAP groups are shown in Additional file 2: Figure S2. There was no difference in prevalence of AKI based on noradrenaline use in the low- and high-MAP groups (*p* = 0.22).

### Time to acute kidney injury

In the unadjusted Cox regression analyses, age, lack of bystander CPR, non-shockable initial rhythm, and time to ROSC were associated with time to AKI KDIGO 1–3 (Table 2) and AKI KDIGO 2–3 (Additional file 10: Table S4) during the first five days after OHCA, whereas MAP was not. In the multivariate Cox regression model, hypertension (HR 1.72; 95% CI 1.07–2.78; p = 0.03), lack of bystander CPR (HR 2.19 (1.38–3.48); *p* < 0.01), non-shockable initial rhythm (HR 2.78 (1.65–4.68); *p* < 0.01), and time to ROSC (HR 1.04; 95% CI 1.02–1.06; *p* < 0.01) were associated with time to AKI KDIGO 1–3, whereas age and MAP were not (Table 2). Similar results were obtained in a Cox regression analyses for time to AKI defined as AKI KDIGO 1–3 focusing on the first 48 h (Additional file 9: Table S3). When using the same covariates, age, lack of bystander CPR, non-shockable rhythm, and time to ROSC were independently associated with time to AKI KDIGO 2–3, whereas previous hypertension and MAP were not (Additional file 10: Table S4).

**Table 2:** Cox proportional hazards regression analysis for time to acute kidney injury (AKI) defined as any KDIGO class 1–3 during the first five days in the ICU and multivariate competing risk analysis when outcome is AKI 1–3 and death

	Univariate HR (95% CI)	*P* value	Multivariate HR (95% CI)	*P* value	Multivariate CR HR (95% CI) Outcome: AKI 1–3	*P* value	Multivariate CR HR (95% CI) Outcome: death	*P* value
Age	1.03 (1.01–1.05)	**0.02**	1.02 (1.00–1.04)	0.06	1.02 (1.00–1.05)	0.06	1.02 (1.00–1.04)	0.11
Lack of bystander CPR	2.59 (1.68–3.98)	** < 0.01**	2.19 (1.38–3.48)	** < 0.01**	2.42 (1.49–3.95)	** < 0.01**	2.19 (1.38–3.48)	0.85
Initial rhythm, non-shockable	2.91 (1.82–4.64)	** < 0.01**	2.78 (1.65–4.68)	** < 0.01**	2.76 (1.60–4.78)	** < 0.01**	2.78 (1.65–4.68)	**0.02**
HTA	1.96 (1.26–3.05)	**0.03**	1.72 (1.07–2.78)	**0.03**	1.71 (1.03–2.83)	**0.04**	1.72 (1.07–2.78)	0.14
Time to ROSC	1.03 (1.00–1.05)	**0.02**	1.04 (1.02–1.06)	** < 0.01**	1.04 (1.02–1.07)	** < 0.01**	1.04 (1.02–1.06)	0.42
MAP high	0.87 (0.57–1.33)	0.51	0.78 (0.50–1.23)	0.29	0.79 (0.49–1.28)	0.34	0.78 (0.50–1.23)	0.60

A two-sided *p* value < 0.05 was considered statistically significant (in bold)

HR hazard ratio, CI Confidence interval, CR competing risk, MAP mean arterial pressure

A comparable model using admission creatinine as the baseline in the AKI definition resulted in comparable results (Additional file 11: Table S5) and a model built to analyse time to AKI KDIGO 2–3 defined with admission creatinine showed no association between the MAP group and AKI (Additional file 12: Table S6). Finally, comparable results were achieved when the AKI definition included the change in creatinine as well as the daily average urinary output (Additional file 13: Table S7).

In the competitive risk analysis, hypertension (HR 1.71 (95%CI 1.03–2.83); p = 0.04), lack of bystander CPR (HR 2.42 (1.49–3.95); *p* < 0.01), non-shockable initial rhythm (2.76 (1.60–4.78); p < 0.01), and time to ROSC (HR 1.04 (1.02–1.07); *p* < 0.01) were associated with time to AKI KDIGO 1–3, whereas age and MAP were not (Table 2 and Additional file 14: Table S8). When the outcome was death, only non-shockable initial rhythm was significant (HR 2.76 (1.60–4.78); *p* = 0.02) in the competitive risk model within AKI KDIGO 1–3 (Table 2 and Additional file 14: Table S8). Comparable results were associated with AKI KDIGO 2–3 (Additional file 14: Table S8).

### MAP and mortality

Mortality was not different in the two studies: 46 patients (41%) within the high-MAP group vs 45 patients (39%) in the low-MAP group (*p* = 0.77).

The median time to death in the lower MAP group during the first 5 days in the ICU was 4.0 (median IQR 3.0–4.5) days in the low-MAP group vs 4.0 (median IQR 3.0–5.0) days in the higher MAP group (*p* = 0.56). A comparison of the mortality at different time-points in low- and high-MAP cohorts is shown in Additional file 15: Table S9.

In addition, we analysed days alive without AKI. Within all patients, median days alive without AKI 1–3 were 3 (IQR 1–4) and without AKI 2–3 were 4 (IQR 2–5). Days alive without AKI 1–3 were 3 (IQR 1–4) in the low-MAP group and 3 (IQR 2–4) in the high-MAP group (*p* = 0.26). Days alive without AKI 2–3 were 4 (IQR 2–5) in the low-MAP group and 4 (IQR 2–5) in the high-MAP group (*p* = 0.82).

### Creatinine and urine output in patients with a history of hypertension

When comparing creatinine values and UO in patients with or without pre-existing hypertension, UO in patients with hypertension was higher in the high-MAP group (*p* = 0.01) (Additional file 3: Figure S3). In the patients without a history of hypertension, no such differences were seen (Additional file 4: Figure S4). We also compared creatinine clearance with CKD- EPI equation and found no statistically significant differences in patients with or without hypertension (Additional file 6: Figure S6).

## Discussion

In this post hoc analysis of two multicentre randomized trials, we assessed the incidence of AKI and compared it between OHCA patients allocated to treatment according to either low-normal or high-normal MAP target. Categorizing AKI by comparing creatinine levels to age- and gender-matched normal levels indicated that AKI occurs in four out of ten OHCA patients and half of the cases were mild (AKI KDIGO 1). AKI was more common in elderly patients. We found no difference in the development of AKI between patients treated with a higher MAP target and those treated with a lower MAP target over the first 36 h. This finding persisted despite several sensitivity analyses with the categorisation of AKI based on actual admission creatinine values, with the inclusion of low daily urinary output in defining more severe AKI or with estimating creatinine clearance using CKD-EPI equation. We did, however, find that in patients with chronic hypertension, a higher MAP target increased the urinary output.

Current guidelines on post-CA care suggest maintaining an MAP of at least 65 mmHg to maintain adequate organ perfusion (indicated by decreasing lactate and adequate UO) by the administration of fluids and norepinephrine with or without inotropes [27]. Previously, a few studies have assessed the relationship between MAP levels and the progression of AKI after OHCA. In the Targeted Temperature Management (TTM)-trial, mean MAP < 70 mmHg compared to 70–80 mmHg and > 80 mmHg was associated with decreased renal function measured by eGFR calculated using the Cockcroft–Gault formula [28, 29]. Another post hoc study of the TTM-trial suggested that patients with higher MAP (4 mmHg), higher heart rate (11 beats/min), and higher lactate had the highest incidence of AKI, whereas there was no difference in cardiac output [30]. A recent study showed that time spent under MAP thresholds 65 to 85 mmHg for the first 6 and 12 h after ICU admission was associated with severe AKI defined as a stage 3 of AKI KDIGO after OHCA, and the highest adjusted odds ratios were observed within 6 h of ICU admission [31]. Hence, Dupont and coauthors suggested that in OHCA patients, targeting MAP as high as 85 mmHg within the first 6 and 12 after ICU admission could decrease the incidence of severe AKI [31]. However, in our study, there was no statistical difference in the incidence of AKI defined as AKI KDIGO stage 1 or stages 2–3 between a low-normal and high-normal MAP groups, suggesting that there are other factors affecting renal injury after CA. The recently published BOX trial that compared in a blinded setup, an MAP target of either 63 mmHg to 77 mmHg found no difference in the need to renal replacement therapy in the two treatment groups [32]. Our recent systematic review of trials targeting a higher or lower MAP after OHCA concluded that the amount of patients included can exclude treatment effects of 25% or higher on mortality and unfavorable neurologic recovery [18]. Whether smaller effects exist should be tested in future larger studies with more heterogeneous patient samples.

In healthy individuals, renal and cerebral blood flow is stable due to autoregulation within a wide range of MAP. During the early stages of shock, renal autoregulation may be temporarily disturbed; thus, patients might benefit from higher levels of MAP to maintain normal renal function. Interestingly, our results showed that lower MAP goals were not associated with higher rates of AKI, suggesting that renal autoregulation is maintained once MAP goals around 65 mmHg are achieved. The association of MAP levels and AKI has been also analysed in other forms of critical illness. In septic shock patients, Asfar et al. found no difference in the incidence of severe AKI staged as AKI KDIGO 2–3 defined as doubling of plasma creatinine (38.7% vs 41.5%, p = 0.42) or need for RRT (33.5% vs. 35.8%, p = 0.5) between a lower and a higher MAP group (65–75 mmHg vs 80–85 mmHg) [33]. They did, however, find that in patients with chronic hypertension, a higher MAP target resulted in less stage 2 AKI KDIGO (38.9% vs 52%, p = 0.02). On the contrary, Poukkanen et al. found that sepsis patients who progressed to develop AKI had lower time-adjusted MAP (74 mmHg) than the patients who did not develop AKI (MAP 79 mmHg) [34]. LeDoux et al. found no significant difference in UO when MAP was raised from 65 to 85 mmHg; therefore, an MAP of 65 mmHg was thought to be adequate to maintain renal perfusion [35]. In a study that randomized patients with intracerebral hemorrhage into lower and higher systolic blood pressure (SBP) groups (110–139 mmHg or 140–179 mmHg), the incidence of renal adverse events was higher in the low SBP group (9.0% vs 4.0%, *p* = 0.002) [36]. One other concern with targeting a higher MAP in critically ill patients has been the possible side-effects of the used vasopressors. In a recent study conducted in patients after cardiac surgery, exposure to norepinephrine was associated with the development of AKI [37]. In the current study, we found no significant difference in the prevalence of AKI based on the needed noradrenaline dose, but we may have been limited by sample size.

Previous studies have shown that risk factors like the presence of chronic hypertension, congestive heart failure, chronic renal disease, age, sex, time to ROSC, total epinephrine dose given during CPR, and a cumulative positive fluid balance may be associated with AKI in patients after CA [3, 6, 7, 38]. In OHCA patients, CA in a public setting and a shockable rhythm have, on the other hand, been suggested as protective factors compared to an unwitnessed arrest and non-shockable initial rhythms [7, 38]. In our study, age, lack of bystander CPR, non-shockable initial rhythm, hypertension, and time to ROSC were independent risk factors for AKI in COX regression. However, as the COMACARE study excluded patients older than 80 and patients resuscitated from a non-shockable rhythm, smaller but clinically relevant effects of higher MAP on renal outcome may exist. Many OHCA patients commonly undergo CAG and PCI, and the use of contrast agents has been feared as a risk factor for AKI. However, in our patient cohorts, contrast administration during angiography was not associated with higher incidence of AKI in the univariate model. In addition, our study is in line with some recent studies showing that early CAG/PCI does not increase the incidence of AKI after CA [39–41]. In the Coronary Angiography after Cardiac arresT (COACT) trial, the incidence of AKI did not differ between early vs late CAG/PCI in patients with non-STEMI [42].

In this post hoc study, the incidence of AKI was higher when the MDRD estimated creatinine level was used as the baseline [11]. When the actual admission creatinine values were used as baseline, the incidence was slightly lower. In the previous studies, the incidence of AKI after CA varies from 37 to 81% [3].

## Strengths and limitations

The current study is a post hoc analysis of two randomized trials, COMACARE and Neuroprotect, in which OHCA patients were randomized into a low-normal or a high-normal MAP group. This enabled us to compare the development of AKI between patients in these two MAP target groups. This extended period of recording is likely to reliably capture changes in creatinine levels and UO and thus follow the development of AKI during the post-resuscitation phase. We acknowledge some limitations in this study. First, we had limited data on several variables potentially relevant to the development of AKI, such as certain patient comorbidities like congestive heart failure, chronic renal disease, diabetes mellitus, factors during resuscitation including the total epinephrine dose given during CPR, the volumes of iodinated contrast agents used during angiography nor other nephrotoxic agents, and the fluid balance in the ICU. Second, although our data were controlled for known confounders, other confounding factors associated with post-CA syndrome may relate to AKI after CA. Third, we did not exclude patients who possibly died in the ICU during the first five days after OHCA. Hence, some of the patients that did not develop AKI in the ICU possibly died before progression of AKI was identified. However, the main point of this study was to look at time to AKI in the high-normal and low-normal MAP groups. Fourth, previous hypertension was associated with AKI KDIGO 1–3 but not when analyses was limited to the more severe forms of AKI, AKI KDIGO 2–3. As more severe AKI is fairly uncommon after OHCA, it is likely that the lack of difference in KDIGO classes 2–3 is related to the limited sample size. Fifth, in COMACARE trial, we included only patients with shockable initial rhythms (VF or VT), whereas Neuroprotect trial included also non-shockable rhythms (PEA and ASY). Indeed, the prevalence of AKI was different in the two studies. Given this, our sample may not accurately estimate the incidence of AKI on more heterogeneous samples of OHCA patients. Future studies should investigate the effect of higher MAP on renal outcomes in unselected OHCA patients, in elderly patients, those with hypertension and diabetes and those resuscitated from an unshockable cardiac arrest rhythm. Finally, we did not have the baseline pre-disease creatine levels in these patients which would have enabled a more accurate calculation of new AKI after cardiac arrest.

## Conclusion

AKI occurs in four out of ten OHCA patients. Independent risk factors associated with AKI are lack of bystander CPR, non-shockable initial rhythm, chronic hypertension, and time to ROSC. Targeting higher MAP after CA does not appear to influence the development of AKI.

### Supplementary Information

**Additional file 1: Figure S1**. Creatinine and urine output (UO) in the low-normal and high-normal mean arterial pressure target groups during the first five days in the COMACARE (a,c) and Neuroprotect (b,d) trials separately.

**Additional file 2: Figure S2**. Median a) percentage and b) count of patients with AKI KDIGO 0, 1 or 2-3 in the low or high MAP treatment groups receiving low or high doses of norepinephrine (NE). The median cut-off value of NE was 0.08 μg/kg/min in the low MAP group and 0.2 μg/kg/min in the high MAP group, p = 0.22.

**Additional file 3: Figure S3**. Creatinine and urine output (UO) in the low-normal and high-normal mean arterial pressure target groups during the first five days after out-of-hospital cardiac arrest in patients with hypertension. a) Creatinine values of patients with hypertension, b) UO of patients with hypertension.

**Additional file 4: Figure S4**. Creatinine and urine output (UO) in low-normal and high-normal mean arterial pressure target groups during the first five days after out-of-hospital cardiac arrest in patients without hypertension. A) Creatinine values of patients without hypertension; b) UO of patients without hypertension.

**Additional file 5: Figure S5**. Kidney function assessed with creatinine clearance (CKD-EPI) over time in the high-normal and low-normal MAP groups.

**Additional file 6: Figure S6**. Creatinine clearance (CKD-EPI) in low-normal and high-normal mean arterial pressure target groups during the first five days after out-of-hospital cardiac arrest in patients a) with hypertension; b) without hypertension.

**Additional file 7: Table S1**. Inclusion and exclusion criteria of the COMACARE and NEUROPROTECT trials.

**Additional file 8: Table S2**. Comparison of patient characteristics between patients with KDIGO stages, 0, 1 and 2–3 during the first five days after cardiac arrest.

**Additional file 9: Table S3**. Cox proportional hazards regression analysis for time to acute kidney injury (AKI) defined as any KDIGO class 1-3 during the first 48 hours in the ICU.

**Additional file 10: Table S4.** Cox proportional hazards regression analysis for time to acute kidney as KDIGO 2-3 injury during the first five days in the intensive care based on changes in creatinine.

**Additional file 11: Table S5**. Cox proportional hazards regression analysis for time to acute kidney injury (KDIGO 1-3) during the first five days in the ICU when creatinine on hospital admission was used as the baseline.

**Additional file 12: Table S6**. Cox proportional hazards regression analysis for time to acute kidney injury defined KDIGO 2-3 during the first five days in the intensive care using creatinine on hospital admission as the baseline.

**Additional file 13: Table S7**. Cox proportional hazards regression analysis for time to acute kidney injury defined as KDIGO 2-3 during the first five days in the ICU with the AKI definition also including changes in urinary output.

**Additional file 14: Table S8**. Competing risk analysis within AKI KDIGO 1-3 when outcome was a) AKI KDIGO 1-3 or b) death; and within AKI KDIGO 2-3 when outcome was c) AKI KDIGO 2-3 or d) death.

**Additional file 15: Table S9**. ICU- and long-term mortality of patients in the high MAP and low MAP patient cohorts.

## Data Availability

The dataset used and analysed during the current study are available from the corresponding author on reasonable request.

## Abbreviations

AKI: Acute kidney injury
CA: Cardiac arrest
KDIGO: Kidney Disease Global outcomes
MAP: Mean arterial pressure
OHCA: Out-of-hospital cardiac arrest
ROSC: Return of spontaneous circulation
UO: Urine output
