# Supplementary material for: The effect of higher or lower mean arterial pressure on kidney function after cardiac arrest: a post hoc analysis of the COMACARE and NEUROPROTECT trials
Source: Ann Intensive Care. 2023 Nov 21;13:113. doi: 10.1186/s13613-023-01210-0 (PMC10663425; doi:10.1186/s13613-023-01210-0)
Supplement: Supplementary file 1 — Additional file 1: Figure S1. Creatinine and urine output (UO) in the low-normal and high-normal mean arterial pressure target groups during the first five days in the COMACARE (a,c) and Neuroprotect (b,d) trials separately. [file 13613_2023_1210_MOESM1_ESM.docx]

**Additional file Figure S1.** **Creatinine and urine output (UO) in the low-normal and high-normal mean arterial pressure target groups during the first five days in the COMACARE (a,c) and Neuroprotect (b,d) trials separately.**
