# Supplementary material for: The effect of higher or lower mean arterial pressure on kidney function after cardiac arrest: a post hoc analysis of the COMACARE and NEUROPROTECT trials
Source: Ann Intensive Care. 2023 Nov 21;13:113. doi: 10.1186/s13613-023-01210-0 (PMC10663425; doi:10.1186/s13613-023-01210-0)
Supplement: Supplementary file 2 — Additional file 2: Figure S2. Median a) percentage and b) count of patients with AKI KDIGO 0, 1 or 2-3 in the low or high MAP treatment groups receiving low or high doses of norepinephrine (NE). The median cut-off value of NE was 0.08 μg/kg/min in the low MAP group and 0.2 μg/kg/min in the high MAP group, p = 0.22. [file 13613_2023_1210_MOESM2_ESM.docx]

**Additional file Figure S2. Median a) percentage and b) count of patients with AKI KDIGO 0, 1 or 2-3 in the low or high MAP treatment groups receiving low or high doses of norepinephrine (NE). The median cut-off value of NE was 0.08 μg/kg/min in the low MAP group and 0.2 μg/kg/min in the high MAP group, p = 0.22.**

**a)**

**b)**
