# Supplementary material for: The effect of higher or lower mean arterial pressure on kidney function after cardiac arrest: a post hoc analysis of the COMACARE and NEUROPROTECT trials
Source: Ann Intensive Care. 2023 Nov 21;13:113. doi: 10.1186/s13613-023-01210-0 (PMC10663425; doi:10.1186/s13613-023-01210-0)
Supplement: Supplementary file 3 — Additional file 3: Figure S3. Creatinine and urine output (UO) in the low-normal and high-normal mean arterial pressure target groups during the first five days after out-of-hospital cardiac arrest in patients with hypertension. a) Creatinine values of patients with hypertension, b) UO of patients with hypertension. [file 13613_2023_1210_MOESM3_ESM.docx]

**Additional file Figure S3.** **Creatinine and urine output (UO) in the low-normal and high-normal mean arterial pressure target groups during the first five days after out-of-hospital cardiac arrest in patients with hypertension. a) Creatinine values of patients with hypertension, b) UO of patients with hypertension.**

MAP mean arterial pressure.
