# Supplementary material for: The effect of higher or lower mean arterial pressure on kidney function after cardiac arrest: a post hoc analysis of the COMACARE and NEUROPROTECT trials
Source: Ann Intensive Care. 2023 Nov 21;13:113. doi: 10.1186/s13613-023-01210-0 (PMC10663425; doi:10.1186/s13613-023-01210-0)
Supplement: Supplementary file 5 — Additional file 5: Figure S5. Kidney function assessed with creatinine clearance (CKD-EPI) over time in the high-normal and low-normal MAP groups. [file 13613_2023_1210_MOESM5_ESM.docx]

**Additional file Figure 5. Kidney function assessed with creatinine clearance (CKD-EPI) over time in the high-normal and low-normal MAP groups.**
