# Supplementary material for: The effect of higher or lower mean arterial pressure on kidney function after cardiac arrest: a post hoc analysis of the COMACARE and NEUROPROTECT trials
Source: Ann Intensive Care. 2023 Nov 21;13:113. doi: 10.1186/s13613-023-01210-0 (PMC10663425; doi:10.1186/s13613-023-01210-0)
Supplement: Supplementary file 7 — Additional file 7: Table S1. Inclusion and exclusion criteria of the COMACARE and NEUROPROTECT trials. [file 13613_2023_1210_MOESM7_ESM.docx]

**Additional file Table 1. Inclusion and exclusion criteria of the COMACARE and NEUROPROTECT trials.**

|  | COMACARE | NEUROPROTECT |
| --- | --- | --- |
| Inclusion criteria | | |
| OHCA | + | + |
| GCS | M < 5 | < 8 |
| Initial rhythm | VF, VT | VF, VT, ASY, PEA |
| Time to ROSC | < 45 min | All patients |
| Witnessed | + | +/- |
| Exclusion criteria | | |
| Suspected intracranial bleeding | + | + |
| Suspected stroke | + | + |
| Pregnancy | + | + |
| Age | < 18 and > 80 yrs | < 18 yrs |
| Other | PaO_2_/FiO_2_ < 100 mmHg | VA ECMO |
|  |  | Open chest |
|  |  | Pacemaker/ICD |
| Treatment group | | |
| MAP intervention group | 80-100 mmHg | 85–100 mmHg |
| SVO_2_ intervention group | - | 65–75% |
| MAP control group | > 65 mmHg | > 65 mmHg |
| PaCO_2_ | 4.5–4.7 kPa or 5.8–6.0kPa | - |
| PaO_2_ | 10–15 kPa or 20–25 kPa | - |

OHCA out-of-hospital cardiac arrest, GCS Glasgow Coma Scale, VF ventricular fibrillation, VT ventricular tachycardia, ASY asystole, PEA pulseless electrical activity, ROSC return of spontaneous circulation, PaO_2_ arterial oxygen partial pressure, FiO_2_ fractional inspired oxygen, VA ECMO veno-arterial extracorporeal membrane oxygenation, ICD implantable cardioverter-defibrillator, MAP mean arterial pressure, SVO_2_ mixed venous oxygen saturation; PaCO_2_ partial pressure of carbon dioxide.
