# Supplementary material for: The effect of higher or lower mean arterial pressure on kidney function after cardiac arrest: a post hoc analysis of the COMACARE and NEUROPROTECT trials
Source: Ann Intensive Care. 2023 Nov 21;13:113. doi: 10.1186/s13613-023-01210-0 (PMC10663425; doi:10.1186/s13613-023-01210-0)
Supplement: Supplementary file 8 — Additional file 8: Table S2. Comparison of patient characteristics between patients with KDIGO stages, 0, 1 and 2–3 during the first five days after cardiac arrest. [file 13613_2023_1210_MOESM8_ESM.docx]

**Additional file Table 2. Comparison of patient characteristics between patients with KDIGO stages, 0, 1 and 2–3 during the first five days after cardiac arrest.**

|  | Data available  227 | All patients | KDIGO 0  n = 145 | KDIGO 1  n = 46 | KDIGO 2–3  n = 40 | p-value |
| --- | --- | --- | --- | --- | --- | --- |
| Sex, n (%) (male) | 227 | 176 (77.5) | 114 (79) | 39 (85) | 25 (63) | **0.03** |
| Age, years (IQR) | 227 | 63 (54–72) | 61 (52–69) | 67 (55–73) | 69 (59–80) | **< 0.01** |
| BMI (IQR) | 209 | 23 (21–25) | 23 (21–25) | 24 (22–26) | 23 (22–26) | 0.18 |
| Cardiac arrest characteristics and resuscitation factors |  |  |  |  |  |  |
| Bystander CPR or  compressions only, n (%) | 221 | 159 (72) | 115 (82) | 28 (62) | 18 (46) | **< 0.01** |
| Public place, n (%) | 223 | 111 (50) | 77 (55) | 20 (49) | 14 (35) |  |
| Initial rhythm | 226 |  |  |  |  | **< 0.01** |
| VF, n (%) |  | 183 (81) | 128 (89) | 37 (80) | 21 (53) |  |
| VT, n (%) |  | 7 (3) | 4 (3) | 1 (2) | 2 (5) |  |
| PEA, n (%) |  | 7 (3) | 2 (1) | 0 (0) | 5 (13) |  |
| ASY, n (%) |  | 29 (13) | 10 (7) | 8 (17) | 12 (30) |  |
| Time to ROSC, minutes  (IQR) | 222 | 20 (14–25) | 18 (14–25) | 20 (14-25) | 21 (15–39) | 0.11 |
| Medical history |  |  |  |  |  |  |
| HTA, n (%) | 221 | 106 (48) | 58 (41) | 30 (70) | 21 (53) | **< 0.01** |
| Diabetes, n (%) | 104 | 10 (10) | 3 (6) | 4 (14) | 5 (16) | 0.10 |
| COPD/ asthma, n (%) | 226 | 20 (9) | 12 (8) | 6 (13) | 6 (15) | 0.10 |
| Betablockers, n (%) | 217 | 64 (30) | 34 (25) | 19 (44) | 13 (35) | **0.02** |
| Ca blockers, n (%) | 217 | 33 (15) | 16 (12) | 9 (21) | 8 (22) | 0.17 |
| ACE inhibitors, n (%) | 215 | 68 (32) | 40 (29) | 20 (48) | 10 (27) | **0.04** |
| Norepinephrine at admission, (IQR) | 209 | 0.13 (0.10-0.24) | 0.13 (0.10-0.24) | 0.18 (0.09-0.27) | 0.13 (0.10-0.24) | 0.74 |
| Treatment |  |  |  |  |  |  |
| CAG | 223 | 187 (84%) | 121 (85) | 39 (87) | 30 (75) | 0.24 |
| PCI | 224 | 112 (50%) | 73 (51) | 26 (58) | 16 (40) | 0.25 |
| MAP treatment group | 227 |  |  |  |  | 0.63 |
| High-normal, n (%) |  | 112 (49) | 74 (66) | 23 (21) | 17 (15) |  |
| Low-normal, n (%) |  | 115 (51) | 71 (62) | 23 (20) | 23 (20) |  |
| Duration of mechanical ventilation, days | 195 | 4 (2–7) | 3.5 (2.1–6.0) | 5.9 (2.9–8.0) | 6.0 (3.0–10.0) | **0.04** |
| RRT, n (%) | 226 | 3 (1.7) | 0 (0) | 0 (0) | 3 (7.5) | **0.01** |
| Length of stay in ICU, days | 221 | 6 (4–9) | 5.7 (3.7–9.1) | 6.0 (4.1–12.8) | 7.0 (3.0–14.0) | 0.22 |
| Death in ICU, n (%) | 227 | 91 (40%) | 40 (28) | 21 (51) | 28 (70) | **<0.01** |
| Death in hospital, n (%) *  (Neuroprotect trial) | 106 | 60 (57) | 20 (42) | 16 (55) | 26 (79) | **<0.01** |
| Mortality 30d, n (%) | 227 | 93 (41%) | 42 (29) | 20 (49) | 29 (73) | **<0.01** |
| CPC 6 months, poor, n (%) | 227 | 106 (47) | 50 (35) | 27 (59) | 32 (80) | **< 0.01** |

BMI body mass index, CPR cardiopulmonary resuscitation, VF ventricular fibrillation, VT ventricular tachycardia, PEA pulseless electrical activity, ASY asystole, ROSC return of spontaneous circulation, HTA arterial hypertension, COPD chronic obstructive pulmonary disease, ACE angiotensin-converting enzyme, CAG coronary angiography, PCI percutaneous coronary intervention, MAP mean arterial pressure, ICU intensive care unit, CPC cerebral performance category, RRT Renal replacement therapy. *”Death in hospital”- data was available on Neuroprotect trial.
