# Supplementary material for: The effect of higher or lower mean arterial pressure on kidney function after cardiac arrest: a post hoc analysis of the COMACARE and NEUROPROTECT trials
Source: Ann Intensive Care. 2023 Nov 21;13:113. doi: 10.1186/s13613-023-01210-0 (PMC10663425; doi:10.1186/s13613-023-01210-0)
Supplement: Supplementary file 9 — Additional file 9: Table S3. Cox proportional hazards regression analysis for time to acute kidney injury (AKI) defined as any KDIGO class 1-3 during the first 48 hours in the ICU. [file 13613_2023_1210_MOESM9_ESM.docx]

**Additional file Table S3. Cox proportional hazards regression analysis for time to acute kidney injury (AKI) defined as any KDIGO class 1-3 during the first 48 hours in the ICU**

|  | Univariate HR  (95% CI) | p-value | Multivariate HR  (95% CI) | p-value |
| --- | --- | --- | --- | --- |
| Age | 1.03 (1.01–1.05) | **< 0.01** | 1.02 (1.00–1.04) | 0.04 |
| Lack of bystander CPR | 2.49 (1.56–3.96) | **< 0.01** | 2.13 (1.31–3.49) | **< 0.01** |
| Initial rhythm, non-shockable | 2.77 (1.66–4.61) | **< 0.01** | 2.62 (1.50–4.59) | **0.04** |
| HTA | 1.94 (1.21–3.10) | **< 0.01** | 1.72 (1.03-2.88) | **0.04** |
| Time to ROSC | 1.03 (1.01-1.05) | **0.02** | 1.04 (1.01-1.06) | **0.02** |
| MAP high | 0.80 (0.51–1.27) | 0.35 | 0.75 (0.46–1.22) | 0.24 |
