# Supplementary material for: The effect of higher or lower mean arterial pressure on kidney function after cardiac arrest: a post hoc analysis of the COMACARE and NEUROPROTECT trials
Source: Ann Intensive Care. 2023 Nov 21;13:113. doi: 10.1186/s13613-023-01210-0 (PMC10663425; doi:10.1186/s13613-023-01210-0)
Supplement: Supplementary file 10 — Additional file 10: Table S4. Cox proportional hazards regression analysis for time to acute kidney as KDIGO 2-3 injury during the first five days in the intensive care based on changes in creatinine. [file 13613_2023_1210_MOESM10_ESM.docx]

**Additional file Table S4.** **Cox proportional hazards regression analysis for time to acute kidney as KDIGO 2-3 injury during the first five days in the intensive care based on changes in creatinine.**

|  | Univariate HR  (95% CI) | p-value | Multivariate HR  (95% CI) | p-value |
| --- | --- | --- | --- | --- |
| Age | 1.05 (1.02–1.08) | **0.02** | 1.04 (1.01-1.07) | **< 0.01** |
| Lack of bystander CPR | 3.34 (1.78–6.28) | **< 0.01** | 2.50 (1.31-4.77) | **< 0.01** |
| Initial rhythm, nonshockable | 5.07 (2.70–9.53) | **< 0.01** | 5.05 (2.52-10.15) | **< 0.01** |
| HTA | 1.26 (0.68-2.35) | 0.46 | 1.03 (0.52-2.03) | 0.93 |
| Time to ROSC | 1.05 (1.03-1.08) | **< 0.01** | 1.07 (1.03-1.10) | **< 0.01** |
| MAP high | 0.73 (0.39–1.36) | 0.32 | 0.68 (0.35–1.31) | 0.25 |

HR hazard ratio, MAP mean arterial pressure
