# Supplementary material for: The effect of higher or lower mean arterial pressure on kidney function after cardiac arrest: a post hoc analysis of the COMACARE and NEUROPROTECT trials
Source: Ann Intensive Care. 2023 Nov 21;13:113. doi: 10.1186/s13613-023-01210-0 (PMC10663425; doi:10.1186/s13613-023-01210-0)
Supplement: Supplementary file 11 — Additional file 11: Table S5. Cox proportional hazards regression analysis for time to acute kidney injury (KDIGO 1-3) during the first five days in the ICU when creatinine on hospital admission was used as the baseline. [file 13613_2023_1210_MOESM11_ESM.docx]

**Additional file Table S5. Cox proportional hazards regression analysis for time to acute kidney injury (KDIGO 1-3) during the first five days in the ICU when creatinine on hospital admission was used as the baseline.**

|  | Univariate HR  (95% CI) | p-value | Multivariate HR  (95% CI) | p-value |
| --- | --- | --- | --- | --- |
| Age | 1.02 (1.00-1.04) | **0.03** | 1.01 (0.99-1.03) | 0.80 |
| Lack of bystander CPR | 2.93 (1.82-4.72) | **<0.01** | 2.72 (1.64-4.54) | **<0.01** |
| Initial rhythm, non-shockable | 1.88 (1.08-3.25) | **0.03** | 1.78 (0.98-3.23) | 0.05 |
| HTA | 1.62 (0.99-2.65) | 0.06 | 1.59 (0.94-2.66) | 0.08 |
| Time to ROSC | 1.04 (1.01-1.06) | **<0.01** | 1.04 (1.04-1.07) | **<0.01** |
| MAP high | 0.89 (0.56-1.44) | 0.65 | 0.79 (0.48-1.29) | 0.35 |
