# Supplementary material for: The effect of higher or lower mean arterial pressure on kidney function after cardiac arrest: a post hoc analysis of the COMACARE and NEUROPROTECT trials
Source: Ann Intensive Care. 2023 Nov 21;13:113. doi: 10.1186/s13613-023-01210-0 (PMC10663425; doi:10.1186/s13613-023-01210-0)
Supplement: Supplementary file 12 — Additional file 12: Table S6. Cox proportional hazards regression analysis for time to acute kidney injury defined KDIGO 2-3 during the first five days in the intensive care using creatinine on hospital admission as the baseline. [file 13613_2023_1210_MOESM12_ESM.docx]

**Additional file Table S6.** **Cox proportional hazards regression analysis for time to acute kidney injury defined KDIGO 2-3 during the first five days in the intensive care using creatinine on hospital admission as the baseline.**

|  | Univariate HR  (95% CI) | p-value | Multivariate HR  (95% CI) | p-value |
| --- | --- | --- | --- | --- |
| Age | 1.02 (1.00-1.04) | 0.07 | 0.98 (0.95-1.01) | 0.12 |
| No bystander CPR | 4.81 (1.61-14.35) | **0.04** | 4.97 (1.63-15.13) | **<0.01** |
| Initial rhythm, non-shockable | 2.14 (0.67-6.84) | 0.05 | 2.78 (0.80-9.71) | 0.05 |
| HTA | 1.54 (0.94-2.50) | 0.08 | 1.29 (0.78-9.71) | 0.64 |
| Time to ROSC | 1.04 (1.02-1.06) | **<0.01** | 1.10 (1.05-1.15) | **<0.01** |
| MAP high | 0.75 (0.26-2.16) | 0.60 | 0.62 (0.21-1.87) | 0.40 |
