# Supplementary material for: The effect of higher or lower mean arterial pressure on kidney function after cardiac arrest: a post hoc analysis of the COMACARE and NEUROPROTECT trials
Source: Ann Intensive Care. 2023 Nov 21;13:113. doi: 10.1186/s13613-023-01210-0 (PMC10663425; doi:10.1186/s13613-023-01210-0)
Supplement: Supplementary file 13 — Additional file 13: Table S7. Cox proportional hazards regression analysis for time to acute kidney injury defined as KDIGO 2-3 during the first five days in the ICU with the AKI definition also including changes in urinary output. [file 13613_2023_1210_MOESM13_ESM.docx]

**Additional file Table S7. Cox proportional hazards regression analysis for time to acute kidney injury defined as KDIGO 2-3 during the first five days in the ICU with the AKI definition also including changes in urinary output.**

|  | Univariate HR  (95% CI) | p-value | Multivariate HR  (95% CI) | p-value |
| --- | --- | --- | --- | --- |
| Age | 1.04 (1.02-1.08) | **<0.01** | 1.04 (1.01-1.07) | **0.01** |
| No bystander CPR | 3.47 (1.83-6.58) | **<0.01** | 2.21 (1.12-4.34) | **0.02** |
| Initial rhythm, non-shockable | 4.49 (2.37-8.52) | **<0.01** | 4.82 (2.29-10.15) | **<0.01** |
| HTA | 1.19 (0.64-2.23) | 0.59 | 1.15 (0.58-2.30) | 0.69 |
| Time to ROSC | 1.04 (1.01-1.07) | **<0.01** | 1.06 (1.03-1.10) | **<0.01** |
| MAP high | 0.62 (0.33-1.17) | 0.14 | 0.67 (0.35-1.28) | 0.22 |
