# Supplementary material for: The effect of higher or lower mean arterial pressure on kidney function after cardiac arrest: a post hoc analysis of the COMACARE and NEUROPROTECT trials
Source: Ann Intensive Care. 2023 Nov 21;13:113. doi: 10.1186/s13613-023-01210-0 (PMC10663425; doi:10.1186/s13613-023-01210-0)
Supplement: Supplementary file 14 — Additional file 14: Table S8. Competing risk analysis within AKI KDIGO 1-3 when outcome was a) AKI KDIGO 1-3 or b) death; and within AKI KDIGO 2-3 when outcome was c) AKI KDIGO 2-3 or d) death. [file 13613_2023_1210_MOESM14_ESM.docx]

**Additional file Table S8. Competing risk analysis within AKI KDIGO 1-3 when outcome was a) AKI KDIGO 1-3 or b) death; and within AKI KDIGO 2-3 when outcome was c) AKI KDIGO 2-3 or d) death.**

1. AKI 1-3

Outcome: AKI 1-3

|  | Univariate HR  (95% CI) | p-value | Multivariate CR HR  (95% CI) | p-value |
| --- | --- | --- | --- | --- |
| Age | 1.03 (1.01-1.06) | **0.01** | 1.02 (1.00–1.05) | 0.06 |
| Lack of bystander CPR | 2.75 (1.78-4.25) | **< 0.01** | 2.42 (1.49–3.95) | **< 0.01** |
| Initial rhythm, non-shockable | 2.85 (1.72-4.74) | **< 0.01** | 2.76 (1.60–4.78) | **< 0.01** |
| HTA | 2.04 (1.31-3.17) | **< 0.01** | 1.71 (1.03-2.83) | **0.04** |
| Time to ROSC | 1.03 (1.01-1.05) | **0.01** | 1.04 (1.02-1.07) | **< 0.01** |
| MAP high | 0.91 (0.59-1.40) | 0.67 | 0.79 (0.49–1.28) | 0.34 |

1. Outcome: death

|  | Univariate HR  (95% CI) | p-value | Multivariate CR HR  (95% CI) | p-value |
| --- | --- | --- | --- | --- |
| Age | 1.03 (1.00–1.05) | **0.06** | 1.02 (1.00–1.04) | 0.11 |
| Lack of bystander CPR | 1.22 (0.56-2.66) | 0.62 | 2.19 (1.38–3.48) | 0.85 |
| Initial rhythm, non-shockable | 3.53 (1.83-6.80) | **< 0.01** | 2.78 (1.65–4.68) | **0.02** |
| HTA | 2.39 (1.24-4.60) | **0.01** | 1.72 (1.07-2.78) | 0.14 |
| Time to ROSC | 1.01 (0.97-1.04) | 0.68 | 1.04 (1.02-1.06) | 0.42 |
| MAP high | 1.73 (0.90-3.33) | 0.10 | 0.78 (0.50–1.23) | 0.60 |

1. AKI 2-3

Outcome: AKI 2-3

|  | Univariate HR  (95% CI) | p-value | Multivariate CR HR  (95% CI) | p-value |
| --- | --- | --- | --- | --- |
| Age | 1.05 (1.01-1.09) | **0.01** | 1.05 (1.02-1.08) | **< 0.01** |
| Lack of bystander CPR | 3.46 (1.84-6.49) | **< 0.01** | 2.73 (1.44-5.19) | **< 0.01** |
| Initial rhythm, non-shockable | 5.27 (2.80-9.93) | **< 0.01** | 5.37 (2.70-10.70) | **< 0.01** |
| HTA | 1.29 (0.70-2.39) | 0.42 | 0.92 (0.48-1.75) | 0.79 |
| Time to ROSC | 1.05 (1.02-1.09) | **< 0.01** | 1.07 (1.04-1.11) | **< 0.01** |
| MAP high | 0.73(0.39-1.36) | 0.33 | 0.65 (0.34-1.25) | 0.20 |

1. Outcome: death

|  | Univariate HR  (95% CI) | p-value | Multivariate CR HR  (95% CI) | p-value |
| --- | --- | --- | --- | --- |
| Age | 1.02 (1.00-1.05) | 0.11 | 1.02 (0.99-1.04) | 0.17 |
| Lack of bystander CPR | 1.34 (0.77-2.34) | 0.30 | 1.21 (0.65-2.24) | 0.55 |
| Initial rhythm, non-shockable | 2.43 (1.41-4.21) | **< 0.01** | 2.38 (1.17-4.85) | **0.02** |
| HTA | 2.27 (1.33-3.86) | **< 0.01** | 1.84 (1.02-3.30) | **0.04** |
| Time to ROSC | 1.02 (0.99-1.04) | 0.30 | 1.02 (0.99-1.05) | 0.23 |
| MAP high | 1.31 (0.79-2.18) | 0.29 | 0.95 (0.51-1.75) | 0.86 |
