# Supplementary material for: The effect of higher or lower mean arterial pressure on kidney function after cardiac arrest: a post hoc analysis of the COMACARE and NEUROPROTECT trials
Source: Ann Intensive Care. 2023 Nov 21;13:113. doi: 10.1186/s13613-023-01210-0 (PMC10663425; doi:10.1186/s13613-023-01210-0)
Supplement: Supplementary file 15 — Additional file 15: Table S9. ICU- and long-term mortality of patients in the high MAP and low MAP patient cohorts. [file 13613_2023_1210_MOESM15_ESM.docx]

**Additional file Table S9. ICU- and long-term mortality of patients in the high MAP and low MAP patient cohorts.**

|  | MAP high  n = 112 | MAP low  n = 115 | p-value |
| --- | --- | --- | --- |
| Mortality in ICU, n (%) | 46 (41.1) | 45 (39.1) | 0.77 |
| Mortality in hospital, n (%) | 29 (53.7) | 31 (59.6) | 0.54 |
| Mortality 30d, n (%) | 48 (42.9) | 45 (39.1) | 0.57 |
| Mortality 180d, n (%) | 50 (44.6) | 50 (43.5) | 0.86 |

% within MAP

Death in hospital data available only in the Neuroprotect trial
